# Computational study of β-*N*-acetylhexosaminidase from *Talaromyces flavus*, a glycosidase with high substrate flexibility

**DOI:** 10.1186/s12859-015-0465-8

**Published:** 2015-01-28

**Authors:** Natallia Kulik, Kristýna Slámová, Rüdiger Ettrich, Vladimír Křen

**Affiliations:** 1 Department of Structure and Function of Proteins, Institute of Nanobiology and Structural Biology of GCRC, Academy of Sciences of the Czech Republic, Zamek 136, 37333 Nove Hrady, Czech Republic; 2 Laboratory of Biotransformation, Institute of Microbiology, Academy of Sciences of the Czech Republic, Videnska 1083, 14220 Praha 4, Czech Republic; 3 Faculty of Sciences, University of South Bohemia in Ceske Budejovice, Zamek 136, 37333 Nove Hrady, Czech Republic

**Keywords:** Molecular docking, Substrate specificity, Unnatural substrates, Phylogenetic analysis

## Abstract

**Background:**

β-*N*-Acetylhexosaminidase (GH20) from the filamentous fungus *Talaromyces flavus*, previously identified as a prominent enzyme in the biosynthesis of modified glycosides, lacks a high resolution three-dimensional structure so far. Despite of high sequence identity to previously reported *Aspergillus oryzae* and *Penicilluim oxalicum* β-*N*-acetylhexosaminidases, this enzyme tolerates significantly better substrate modification. Understanding of key structural features, prediction of effective mutants and potential substrate characteristics prior to their synthesis are of general interest.

**Results:**

Computational methods including homology modeling and molecular dynamics simulations were applied to shad light on the structure-activity relationship in the enzyme. Primary sequence analysis revealed some variable regions able to influence difference in substrate affinity of hexosaminidases. Moreover, docking in combination with consequent molecular dynamics simulations of C-6 modified glycosides enabled us to identify the structural features required for accommodation and processing of these bulky substrates in the active site of hexosaminidase from *T. flavus*. To access the reliability of predictions on basis of the reported model, all results were confronted with available experimental data that demonstrated the principal correctness of the predictions as well as the model.

**Conclusions:**

The main variable regions in β-*N*-acetylhexosaminidases determining difference in modified substrate affinity are located close to the active site entrance and engage two loops. Differences in primary sequence and the spatial arrangement of these loops and their interplay with active site amino acids, reflected by interaction energies and dynamics, account for the different catalytic activity and substrate specificity of the various fungal and bacterial β-*N*-acetylhexosaminidases.

**Electronic supplementary material:**

The online version of this article (doi:10.1186/s12859-015-0465-8) contains supplementary material, which is available to authorized users.

## Background

β-*N*-Acetylhexosaminidases (hexosaminidases) belonging to the family 20 of glycoside hydrolases (GH-20; www.cazy.org) are *exo*-glycosidases catalyzing the hydrolysis of terminal nonreducing β-D-GlcNAc and β-D-GalNAc units from a wide variety of glycoconjugates and thus playing an important role in many biological processes [1]. Additionally to their primary hydrolytic activity, these enzymes have been shown to catalyze transglycosylation reactions, where a carbohydrate moiety is transferred from an activated sugar donor to its acceptor, typically an alcohol or a carbohydrate, which makes them a good alternative to glycosyltransferases due to high regioselectivity and lower cost of the substrates [2]. Amongst the hexosaminidase family, the enzymes obtained from filamentous fungi, especially those from the *Aspergillus*, *Penicillium* and *Talaromyces* genera, have proved a great potential in the synthetic reactions, moreover, they have shown enormous substrate flexibility by accepting a variety of unnatural substrates [3-7]. The β-*N*-acetylhexosaminidase from *Talaromyces flavus* CCF2686 has found its prominent position within the fungal enzymes with its extraordinary results in the transglycosylation reactions with the 4-deoxy-substrates [8] and C-6 oxidized and negatively charged substrates [9].

The biochemical properties and structure of β-*N*-acetylhexosaminidase from *Aspergillus oryzae* as the commonly used and commercially available representative of fungal hexosaminidases has been investigated during the last few years in order to reveal the structure-activity relationships in this group of enzymes. The authors found that in fungi the β-*N*-acetylhexosaminidase gene contains a large N-terminal propeptide, which has to be cleaved off by a dibasic peptidase and non-covalently reassociated with the catalytic subunit; the fully active enzyme comprises two catalytic subunits with the two large propeptides attached [9,10]. Even though the crystal structure of a fungal β-*N*-acetylhexosaminidase is of a great interest, neither a resolved structure is published nor released in the protein structure database despite the four years ago reported successful preparation of high-resolution X-ray diffracting crystals of β-*N*-acetylhexosaminidase from *A. oryzae* [11]. To overcome the lack of structural information of fungal β-*N*-acetylhexosaminidase, a homology model of the glycosylated dimeric form of *T. flavus* enzyme was built, and compared to modelled fungal β-*N*-acetylhexosaminidases from *A. oryzae* [12] and *Penicillium oxalicum* [13], correctness of which was validated by biochemical studies and vibrational spectroscopy.

Up to date, most of the reported crystal structures of β-*N*-acetylhexosaminidases originated from bacteria: *Streptomyces plicatus* (1jak) [14], *Paenibacillus* sp. (3gh4) [15], *Streptococcus pneumoniae* (3 rpm) [16], *Streptococcus gordonii* (2epk) [17], *Serratia marcescens* (1qbb) [18], *Actinobacillus actinomycetemcomitans* (1yht) [19] and *Arthrobacter aurescens* (3rcn). Also the structures of α and β chains of human HexA and HexB have been solved [20-23]. More importantly, the chitinolytic hexosaminidase from the moth Asian corn borer *Ostrinia furnacalis* has been recently intensively studied as a potential target for insecticides [24,25] and its structure has been identified as a useful template for the modeling of fungal hexosaminidases.

The common overall protein fold of GH family 20 β-*N*-acetylhexosaminidases is the (β/α)_8_-barrel structure of the catalytic domain housing the active site. The active site contains a highly conserved pair of catalytic residues Asp-Glu, which was proposed shortly after the first crystal structure of a bacterial β-*N*-acetylhexosaminidase with its natural substrate chitobiose bound in its active site was resolved [18]. This enzyme group employs a modified reaction mechanism of retaining glycosidases, which is referred to as substrate-assisted catalysis. In this reaction scheme, the catalytic glutamate acts as a proton donor and the substrate’s 2-acetamido moiety serves as a nucleophile instead of the catalytic aspartate, forming oxazoline reaction intermediate instead of the classical covalent enzyme-substrate complex [26,27].

In this paper, a computational study of β-*N*-acetylhexosaminidase from *Talaromyces flavus* (TfHex), the enzyme with high biotechnological potential in the biosynthesis of unnatural oligosaccharides, whose nucleotide sequence has been determined quite recently [28] is reported. The three-dimensional structure of this interesting enzyme and its comparison with the previously published models of fungal hexosaminidases from *A. oryzae* [12] and *P. oxalicum* [13] and the bacterial crystal structures from *S. plicatus* [14], differing mainly in their affinities towards the C-6 charged substrates [7], reveal the structural features responsible for the observed substrate specificities. Homology modeling together with molecular dynamics simulations was applied to obtain the structure of TfHex useful for the complex description of its enzymatic properties and further determination of the structural basis of its higher affinity to C-6 modified substrates in terms of binding energy and persistence of the interaction. Binding energies of substrates in the active site were estimated with Autodock for initial docked poses as well as for enzyme-substrate complexes resulting from molecular dynamics simulations. Moreover, the molecular dynamics simulations allowed us to study the stability of enzyme-substrate complexes in time and to estimate if substrate not only finds the active site, but also stays bound in a conformation with favorable interaction energy while maintaining essential bonds and a steric arrangement that allows the hydrolysis reaction to proceed. These data represent the real added value that would still have its worth even if a crystal structure of fungal hexosaminidase will be released. Consequently, these data were used to explain results obtained in various wet experiments (reviewed in [29]) to gain a full picture of the structure-activity relationship of unnatural substrates in the active site of the enzyme.

## Results and discussion

### Relationship of the sequence of β-*N*-acetylhexosaminidase from *T. flavus* with hexosaminidases from different organisms

**Figure 1:**
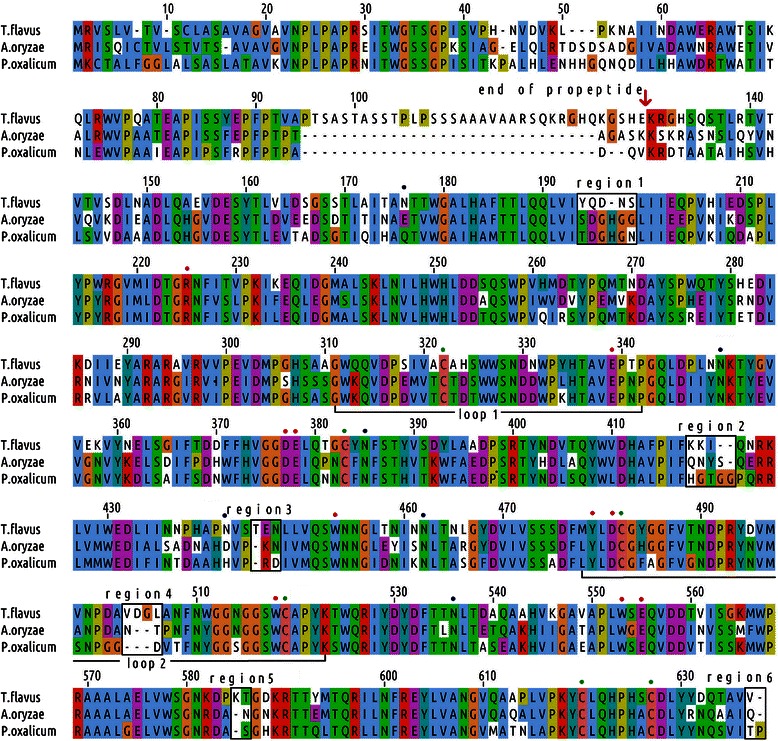
**Multiple sequence alignment of fungal β-** ***N*** **-acetylhexosaminidases.** Cysteine residues are marked by green dots, amino acid residues in *A. oryzae* and *P. oxalicum* active sites are marked by red dots. Long loops close to the active site are labeled. The C-terminal end of the propeptide is marked, insertion/deletion regions in the rest of the protein are shown by black rectangles. ClustalW coloring scheme is used.

The primary sequence of TfHex displayed 83% and less identity with putative hexosaminidases from other *Talaromyces* species, 62% and less with β-*N*-acetylglucosaminidases and β-*N*-acetylhexosaminidases from other fungal genera, 42% and less with some unclassified plant proteins, 36% and less with animal hexosaminidases, 29% and less with bacterial β-*N*-acetylhexosaminidases. The identities of the full sequence of β-*N*-acetylhexosaminidases from *Talaromyces flavus* [GenBank:AEQ33603] with its homologs from *Aspergillus oryzae* [GenBank:AAM13977] and *Penicillium oxalicum* [GenBank:ABY57948] are 61% and 60%, respectively. Multiple sequence alignment of these sequences (Figure 1) shows a large insertion in the propeptide sequence of TfHex before the catalytic domain, however, the three-dimensional structure as well as the orientation of the propeptide in fungal hexosaminidases is not known and it is not possible to estimate its position using the available enzyme templates. The length of the sequences encoding the catalytic and N-terminal domains is similar in the templates with only 6 variable regions of minor insertions or deletions. Apparently, active site amino acids and cysteine residues are conserved.

**Figure 2:**
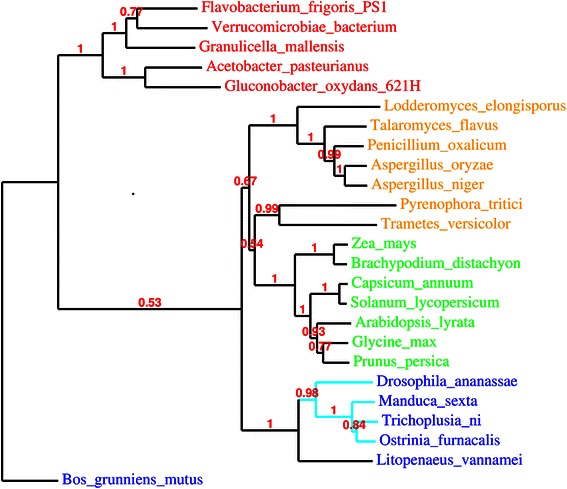
**Phylogram of β-** ***N*** **-acetylhexosaminidases from different organisms.** Names of organisms are colored in groups; each color corresponds to a different kingdom: red – Bacteria, blue – Animalia, green – Plantae, orange – Fungi. The sequence of a single mammalian organism (*Bos grunniens mutus*) is used as an out-group. Cyan branches are used to highlight insect β-*N*-acetylhexosaminidases. Bootstrapping values of branch support are shown over the corresponding branches in red color.

Evolutionarily, TfHex appear closer related to *A. oryzae* and *P. oxalicum* than other fungal hexosaminidase sequences available in the NCBI database (Figure 2). The consensus phylogram using sequences of hexosaminidases from a wide variety of organisms revealed close evolutionary relationship of fungal and plant hexosaminidases (Figure 2).

Interestingly, the sequences of enzymes from *Pyrenophora tritici* and *Trametes versicolor* are even more similar to plant hexosaminidases than to other TfHex-related fungal sequences; however, the bootstrapping demonstrates a rather low probability for this branch. In general, fungal hexosaminidases seem to be evolutionarily closer to plant, insect and whiteleg shrimp (*Litopenaeus vannamei*) enzymes than to those from bacteria and mammals – wild yak (*Bosgrunniens mutus*).

**Figure 3:**
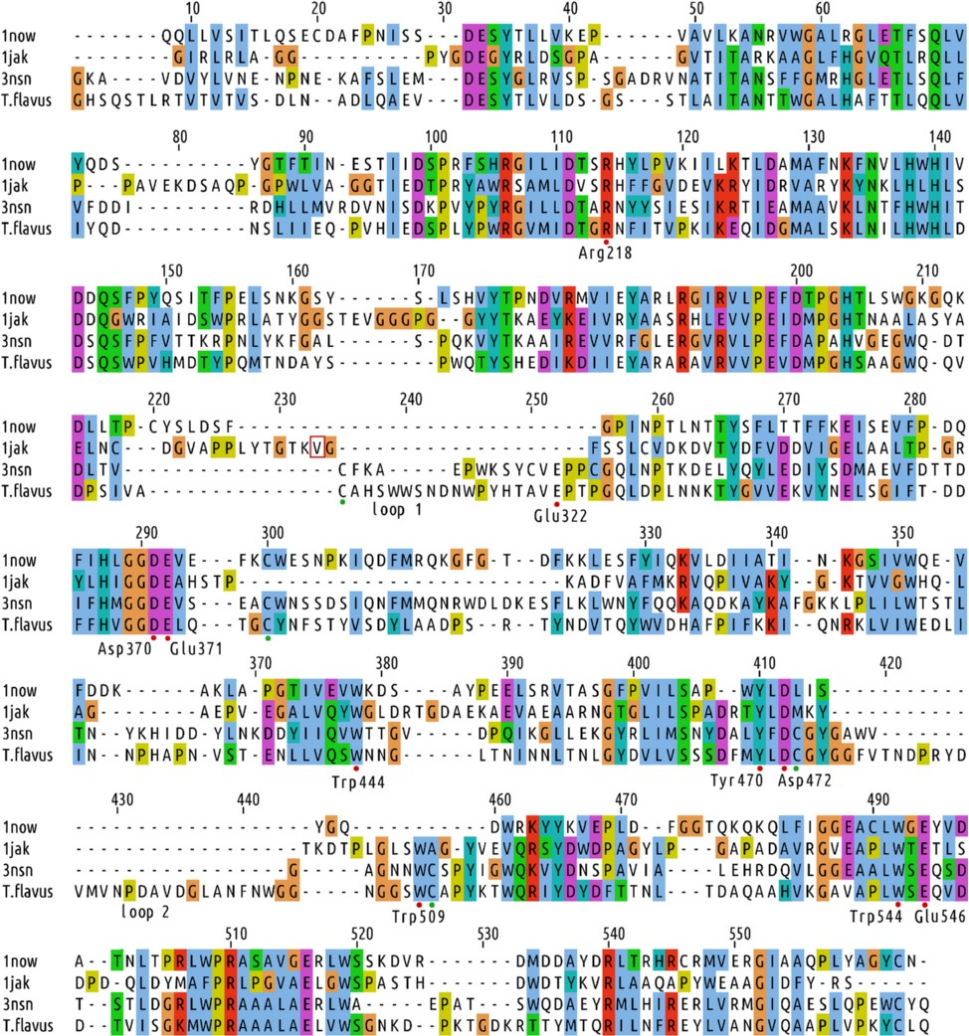
**The multiple sequence alignment used for homology modeling of the TfHex monomer.** Active site amino acids are marked by red dots. Cysteine residues are marked by green dots. Active site amino acids are numbered according to the sequence of β-*N*-acetylhexosaminidase from *T. flavus* (TfHex). 1jak - hexosaminidase from bacteria *Streptomyces plicatus*; 1now - human HexB; 3nsn - hexosaminidase from insect *Ostrinia furnacalis*. Val 276 in *S. plicatus* hexosaminidase is shown by red box.

Results of the BLAST search [30], the multiple sequence alignment and the structural alignment of hexosaminidases revealed that there are two highly diverged regions close to the active site in the catalytic domain of these enzymes, corresponding to loops. These loops feature different length and orientation in the crystal structures of bacterial, human and insect hexosaminidases (Figure 3); the observed differences are not a result of loop flexibility, but rather a structural feature. Thus, we found reasonable to use the results of the phylogenetic analysis of TfHex to guide the refinement of the multiple sequence alignment in highly variable loops and to select the appropriate template for these regions. In hexosaminidases from *A. oryzae* and *P. oxalicum*, the loop 1 is of similar size to TfHex, while loop 2 is shorter in the middle part (Figure 1, Additional file 1: Figures S1-S2). Based on close evolutionary relationship of TfHex with insect enzymes and higher similarity of both loops to insects than to bacterial or mammalian enzymes, these loops were initially modeled based on the insect (3nsn) loop conformation (Figure 3).

### Structural aspects of β-*N*-acetylhexosaminidase from *T. flavus* important for substrate binding

The recently obtained complete sequence of β-*N*-acetylhexosaminidase from *Talaromyces flavus* [28] enabled us to build reliable molecular models of the catalytic subunit of the enzyme as well as models of its dimeric and *N*-glycosylated forms. After extensive sequence and structural alignments, the known three-dimensional structures of hexosaminidases from human (1now), the insect *Ostrinia furnacalis* (3nsn) and the bacterium *Streptomyces plicatus* (1jak) were selected as the most suitable templates for molecular modeling of TfHex. The best models of TfHex built with Modeller [31] were selected for further refinement with molecular dynamics simulation. C-alpha atoms of the best model displayed a long stable RMSD already after first 10 ns of unrestrained refinement run with the RMSD plateau below 0.17 nm over the whole simulation run, corresponding to a well equilibrated model (For more details see Additional file 1: Figure S3).

The averaged secondary structure content during the last 5 ns of simulation is 31.62% of α-helix; 15.2% of β-sheet; 10.9% of turn and the rest – coil. Statistical analysis of the model geometry by Molprobity [32] and Vadar [33] gives the reasonable statistical parameters - 95.88% of protein residues appear in favored region of the Ramachandran plot, only two residues - His 300 and Gly 368 - are found in a disallowed region [32], reflecting some steric problems as a result of poor templates for the loop region following His 300. Energetic parameters of the structural model correspond to typical values found for structures solved by X-ray crystallography (Additional file 1: Figure S3).

**Figure 4:**
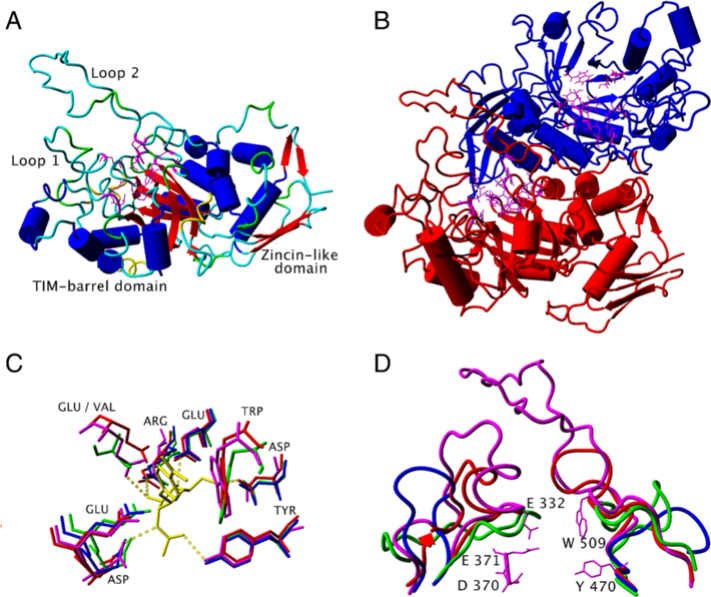
**Model of TfHex. A**. Side view of monomeric TfHex with active site amino acids shown in magenta and stick representation. **B**. Dimeric TfHex. Each monomer is colored by a different color, active site amino acids are shown in magenta. **C**. Overlay of the active site of hexosaminidases from *S. plicatus* (green), *T. flavus* (red), human (blue) and *O. furnacalis* (magenta), the standard substrate is colored in yellow, hydrogen bonds are shown by yellow dotted lines. **D**. Overlay of bacterial *S. plicatus* (green), human (blue), insect *O. furnacalis* (red) hexosaminidases and TfHex (magenta). Loops 1 (left) and 2 (right) are shown in cartoon representation. Active site amino acids of TfHex are shown in stick representation and labeled with one letter code. Glu 332 and Trp 509 belong to loop 1 and 2 correspondingly.

Analogously to the models of hexosaminidases from *A. oryzae* and *P. oxalicum*, the refined model of the TfHex catalytic domain comprises the small *N*-terminal zincin-like domain and the (a/b)_8_ TIM-barrel housing the active site in its center (Figure 4A-B). The amino acids in the active sites of template β-*N*-acetylhexosaminidases are conserved with the exception of the residues corresponding to Glu 332 and Trp 509, which was revealed by the overlay of the active sites of the templates and TfHex with docked *p*NP-GlcNAc (Figure 4C). In TfHex, Glu 332 belongs to loop 1 and occupies the corresponding place in the structure of insect hexosaminidase, while in most of the bacterial hexosaminidase glutamate is substituted by a non-polar residue, such as aliphatic Val 276 in *S. plicatus* (Figures 3 and 4C). The corresponding region of loop 1 has not been resolved in the crystal structure of human hexosaminidase, however, the sequence of the loop contains no Glu or Val residues. The multiple sequence alignment used for phylogenetic analysis revealed high conservation of glutamic acid at the corresponding position in fungal, insect and plant homologs to *T. flavus*, while in bacterial hexosaminidases this residue is mostly substituted by residues with an apolar side chain (Additional file 1: Figure S1-2).

The three cysteine pairs forming disulfide bridges in TfHex are in the same spatial positions in the template enzyme from *O. furnacalis* (Figure 3) and in the fungal homologs (Figure 1): Cys 315-Cys 376 fix the edges of loop 1; Cys 473-Cys 510 fix the *N*-terminal end of loop 2 close to the enzyme active site; Cys 611-Cys 618 connect the catalytic domain to the C-terminal part and has not been modeled, as we found no suitable template for the modeling of the C-terminus (terminal sequence HPHSCDLYYDQTAVV). Six minor variable regions were identified in the multiple sequence alignment of the studied fungal β-*N*-acetylhexosaminidases (Figure 1), however, they are positioned far from the active site and do not contain any residues of the active center or in contact with the substrate.

Modeling of the two long flexible loops positioned above the active site of the enzyme was especially challenging in the case of TfHex, as these loops are even longer than in the other fungal enzymes as shown in the multiple sequence alignment (Figure 1). However, when the structure of the insect hexosaminidase (3nsn) was used as a template, the loop edges could be modeled with sufficient precision. Dimer formation also brings in new information in modeling of loop orientation. Loop 1 (Val 313 - Pro 335) comprises Glu 332 residue of the active site (Figure 4D). Loop 2 containing active site’s Trp 509 is placed above the active site in the inter-monomer surface. This loop is stabilized by interactions with loop 1 and with the other monomer involving hydrogen bonding interactions (residues Asn 418, Arg 484, Gln 517, Thr 577, Asp 579) and π-π stacking interactions (residues Tyr 475 of one monomer and Tyr 513 of another monomer). Arginine 484 of loop 2, which interacts with Asp 579 and Thr 577 from the other monomer, belongs to the fungal variable region 5 (KTGDK in Figure 1). The substitution of tyrosine 475 by histidine in *A. oryzae* hexosaminidase and phenylalanine in *P. oxalicum* hexosaminidase may influence the flexibility of loop 2 and determine the differences in local conformation of fungal β-*N*-acetylhexosaminidases. Loops 1 and 2 are both close to the active site and establish direct contacts with the aglycone part or leaving group of the substrate.

Like in other fungal hexosaminidases, the active site of TfHex is formed by residues of just one monomer (Figure 4) and highly conserved among the studied fungal enzymes (Figure 1)*.* Aspartate 370 and glutamate 371 were identified as the key catalytic residues, while four tryptophan residues (Trp 421, 444, 509 and 544) form a hydrophobic pocket in the active site and participate in stacking interactions with the substrate. Other residues forming hydrogen bonds with the natural substrate chitobiose are Arg 218, Glu 332, Tyr 470, Asp 472, Glu 546 and Trp 509 (Figure 4). Tryptophan 509 forms π-π stacking interaction with +1 sugar of the carbohydrate chain; the leaving group is stabilized not only by stacking with Trp 509, but also by a weak electrostatic interaction with Glu 332 (Figure 4), moreover, some snapshots in molecular dynamics simulations showed also an interaction with Tyr 327 from loop 1.

### Effect of *N*-glycosylation of TfHex on its activity

**Figure 5:**
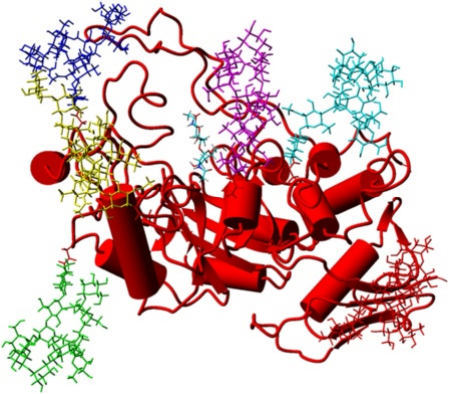
**Glycosylated model of TfHex.** Side view of glycosylated, monomeric TfHex with carbohydrate antennae shown as stick models (red is connected to Asn 170, green – Asn 343, blue – Asn 378, yellow – Asn 433, magenta – Asn 453, cyan – Asn 527). Position of the natural substrate chitobiose is shown in the active site in stick representation colored by element colors.

Six potential *N*-glycosylation sites were identified in the sequence of TfHex by GlyProt [34]: carbohydrate antennae could be attached to asparagine residues 170, 343, 378, 433, 453, 527. Four of the potential *N*-glycosylation sites (378, 343, 527 and 453) correspond to the confirmed *N*-glycosylated sites in both *A. oryzae* [12] and *P. oxalicum* enzymes [13] (Figure 1). For the modeling of the carbohydrate chains a typical glycan – high-mannose oligosaccharide - was employed (LinucsID is 298 in http://www.glycosciences.de/database/index.php); the model of a fully glycosylated monomer of TfHex is shown in Figure 5.

Sugar antennae cover 18.4% of the solvent accessible surface of the modeled enzyme, leading to its decrease of only 2% during molecular dynamics simulation. The total average protein solvent accessible surface calculated by YASARA is similar in both glycosylated and deglycosylated models (the difference was less than 0.6%) and remains within limits proposed for exposition of charged and non-polar residues of globular proteins [35]. The glycan connected to Asn 378 covered the surface of loop 1 and established hydrogen bond interaction with loop 2. However, the study of amino acid deviation close to the mentioned glycan during molecular dynamics did not reveal significant influence of glycosylation on loop stability: the RMSD of amino acid residues of loop 1 in the presence or absence of the sugar chain remained the same and loop 2 was only slightly more flexible in the deglycosylated model (Additional file 1: Figure S4).

Overall, the role of protein *N*-glycosylation in maintaining general protein structure stability or in the protection from solvation seems not to be significant, which had also been observed in the experiments with the deglycosylation of TfHex in our previous work [28]. The modeled glycans occupy space in a sufficient distance from the active site to exclude a major influence on the access or correct binding of the substrates.

### Evidence for different substrate affinity by molecular dynamics simulation of substrates in the active site of β-*N*-acetylhexosaminidases

There is a major interest in the broad substrate specificity of fungal β-*N*-acetylhexosaminidases, which can be applied in the synthesis of a variety of modified glycosides. Besides wet experiments, models of hexosaminidases from *A. oryzae* and *P. oxalicum* were used for studies of interactions of unnatural substrates with these enzymes [7,8,12,13]. Unfortunately, in these earlier studies the primary sequence of the mostly employed and most efficient and flexible β-*N*-acetylhexosaminidase was not known, now this is the first time the enzyme-substrate interactions are reported for the synthetically promising TfHex.

**Figure 6:**
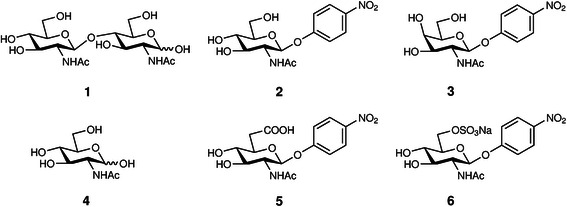
**Structures of ligands docked in the active sites of β-** ***N*** **-acetylhexosaminidases.** Ligands are: **1** – chitobiose; **2** – *p*NP-GlcNAc; **3** – *p*NP-GalNAc; **4** – GlcNAc; **5** – *p*NP-GlcNAc-6-uronate; **6** – *p*NP-GlcNAc-6-sulfate.

For the current study a set of six compounds (Figure 6) was selected for molecular dynamics simulation with hexosaminidases from the fungus *Talaromyces flavus* and from the bacterium *Streptomyces plicatus*, which is one of the first enzymes of this group that has been explored in detail and features a rather narrow substrate flexibility [14] (Table 1). The artificial substrate of β-*N*-acetylhexosaminidases *p*-nitrophenyl 2-acetamido-2-deoxy-β-D-glucosaminide (*p*NP-GlcNAc, 2) has been set as a standard substrate in this work and is used as a reference for the identification of binding affinity and interactions of substrates in the active sites of the enzymes. The other reported compounds are as follows (Figure 6): chitobiose (1, natural substrate of chitinolytic hexosaminidases); *p*NP-GalNAc (3, C-4 epimer of the standard substrate); *N*-acetylglucosamine (4, product of hydrolysis of 1 and 2); *p*NP-GlcNAc-6-uronate (5, C-6 oxidized derivative of 2) and *p*NP-GlcNAc-6-sulfate (6, C-6 negatively charged derivative of 2). The results of the experiments and calculation of the binding energies of equilibrated complexes are presented in Tables 1 and 2, respectively.

**Table 1:** **Relative activity of β-** ***N*** **-acetylhexosaminidases**

**Enzyme source**	**Relative activity (100% corresponds to activity with** ***p*** **NP-GlcNAc), %**		
	***p*** **NP-GalNAc 3**	***p*** **NP-GlcNAc-uronate 5**	***P*** **NP-GlcNAc-sulfate 6**
*Aspergillus oryzae*	56 [36]	2 [7]	<1 [7]
*Penicillium oxalicum*	160 [36]	5 [7]	6 [7]
*Talaromyces flavus*	140	8 [7]	13 [7]
*Streptomyces plicatus*	15	1	4

**Table 2:** **Binding energies of docked compounds**

**Compound**	Binding energies [kcal/mol]*	
	***T. flavus*** **hexosaminidase**	***S. plicatus*** **hexosaminidase**
Chitobiose **1**	−10.83 ± 0.942	−9.51 ± 0.814
*p*NP-GlcNAc **2**	−8.72 ± 0.829	−9.38 ± 0.716
*p*NP-GalNAc **3**	−9.61 ± 1.137	−9.44 ± 0.062
GlcNAc **4**	−6.23 ± 0.305	−7.37 ± 0.386
*p*NP-GlcNAc-uronate **5**	−6.80 ± 0.463	−6.26 ± 0.085
*p*NP-GlcNAc-sulfate **6**	−7.02 ± 0.378	−6.44 ± 0.332

*Binding energy is calculated by AutoDock and represented in the form of average energy for representaive substrate-enzyme complexes and standard deviation.

**Figure 7:**
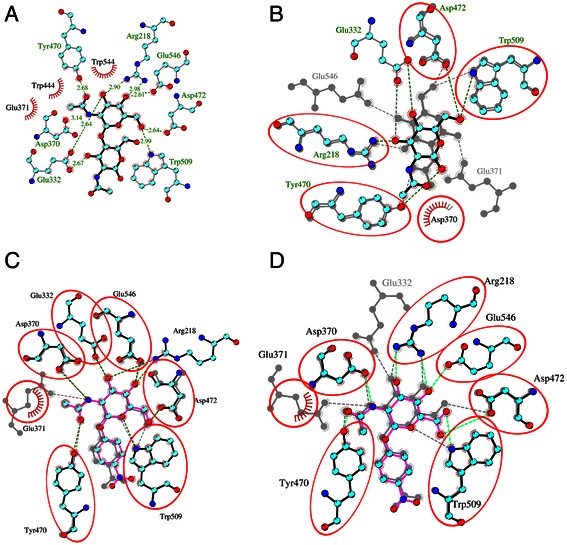
**Substrate dynamics in the active site of TfHex. A**. Active site with docked natural substrate chitobiose **1** after 10 ns of molecular dynamics simulation. **B**. Overlay of the active sites of TfHex with docked GlcNAc (**4**) in the beginning of molecular dynamics (grey) and during stable period (vivid color). Tyr 470, which normally fixes the substrate’s acetamido-group by hydrogen bond with its oxygen, established new interaction with oxygen at C1-atom. **C**. Overlay of *p*NP-GalNAc (**3**, grey) and *p*NP-GlcNAc (**2**, vivid) in the active site of TfHex after 10 ns molecular dynamics*.* Common residues are in red circles, Arg 218 with **3** is not shown. **D**. Overlay of the active sites of TfHex with docked *p*NP-GlcNAc-uronate (**5**; vivid color, yellow color - hydrogen bonds) and *p*NP-GlcNAc (**2**; grey).

The least favorable binding energy obtained with TfHex was observed when docking the product of hydrolysis of chitobiose and *p*NP-GlcNAc – *N*-acetylglucosamine (GlcNAc, 4). Here, the initial docking energy got less favorable by more than 1 kcal/mol during the molecular dynamics simulation. The position of GlcNAc in the active sites of both bacterial and fungal enzymes changed significantly during molecular dynamics, that was accompanied by changes in the hydrogen bonding interactions with the catalytic residues when compared to the natural substrate (Figure 7A-B), so that the position of the catalytic residues after simulations with GlcNAc facilitates the release of the product out of the active site (Additional file 1: Figure S5). The value of the calculated binding energy for the product can be used as a threshold for estimation of successful binding of the substrates, as it is generally accepted that the product should be quickly released from the active site. Moreover, we assume that the behavior of GlcNAc-hexosaminidase complexes during the equilibrated period of the simulation, which is characterized by stable root mean square deviation of C-alpha atoms and interaction energies, can predict the changes occurring in the active site before the departure of the product. In the recently published paper on insect hexosaminidase from *O. furnacalis* [25,37], the ‘open-close’ conformation of the active site during hydrolysis caused by the rotation of catalytic Gly 368 and Trp 448 was proposed. Based on the herein reported molecular dynamics simulations of fungal and bacterial β-*N*-acetylhexosaminidases we can enhance this view by proposing an additional set of changes regulating the product release: rotation of catalytic Glu side chain and shift of Cα-atoms of the catalytic residues, which could regulate the access to the active site.

Binding energies of *T. flavus* β-*N*-acetylhexosaminidase with *p*NP-GalNAc (3) are slightly more favorable than with the *gluco*-configured substrate 2, while for *S. plicatus* enzyme both energies are comparable (see Table 2). As a result of the opposite orientation of the hydroxyl group at C-4 atom, *p*NP-GalNAc lost the persistent interaction with the close-by arginine residue in both hexosaminidases (residues 218 in *T. flavus* and 162 in *S. plicatus* hexosaminidases; Figure 7C). Experimental data show that relative activity of TfHex with *p*NP-GalNAc (3) is higher than with *p*NP-GlcNAc (2), while in the bacterial enzyme the ratio was shifted in favor of the *gluco*-substrate 2. Here, the decrease in activity is not only the result of a differences in binding energy; closer inspection of the interactions of substrate 3 and 2 in the active site revealed that catalytic Glu 314 in *S. plicatus* hexosaminidase lost the stable interaction with O5 atom and changed its orientation in the very beginning of the simulation. As a result, Glu 314 formed an unexpected hydrogen bond with hydroxyl at C3 after 3.7 ns of molecular dynamics, making the first step of hydrolysis impossible to proceed [14,27] (Additional file 1: Figure S6). Finally, all three independent molecular dynamics simulations showed a higher probability of the interaction of Glu 314 in *S. plicatus* hexosaminidase with hydroxyl at C3 atom of compound 3 than an interaction with the oxygen forming glycosidic bond. A representative graph is shown in Additional file 1: Figure S6B. In TfHex, the catalytic Glu 371 kept the hydrogen bond with O5 atom during simulation, because the position of substrate’s C3 in the fungal enzyme is stabilized by Glu 332 from loop 1, which is substituted by the non-polar Val 276 in bacteria (Figures 2 and 7). In summary, the observed divergences in activities of bacterial and fungal hexosaminidases to pNP-GalNAc originated mainly in the primary structure of loop 1, not only in the binding energies.

In our previous work, we have identified the β-*N*-acetylhexosaminidase from *T. flavus* as an enzyme with extremely high substrate flexibility, as it was able to utilize a variety of unnatural substrates including the C-6 oxidized *p*NP-GlcNAc-uronate (5) and C-6 negatively charged *p*NP-GlcNAc-sulfate (6) (Table 1). Finally now, with the model of this enzyme in our hands, we can have a closer look at the interactions of these unnatural substrates with the active site of TfHex. Generally, TfHex binds C-6 modified substrates 5 and 6 with slightly higher affinity than the product 4, which was set as a threshold for successful binding (Table 2). The uronate-bearing substrate 5 is able to form 4–7 hydrogen bonds with TfHex, however, the interactions with Glu 332 and catalytic Glu 371 were not persistent in any of the molecular dynamics runs (Figure 7D). Hereby, an interaction would be considered persistent if present in at least 50% of the snapshots of the equilibrated part of the respective trajectory. Better accommodation of substrate 5 in the active site of TfHex was accompanied by the rotation of side chain of Glu 371 during simulation, which increased the distance of the catalytic Glu residue from the glycosidic bond and prevented effective hydrolysis of the substrate.

**Figure 8:**
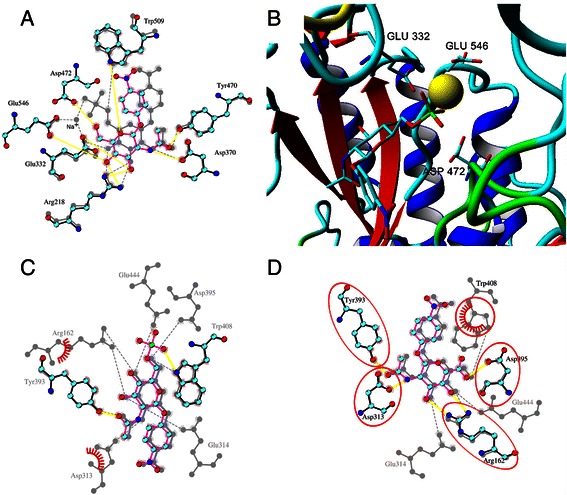
**Docking of C-6 modified substrates. A**. Overlay of the active sites of TfHex with docked *p*NP-GlcNAc (**2**; vivid color, yellow hydrogen bonds) and *p*NP-GlcNAc-sulfate (**6**; grey). Sodium ion in the active site with the sulfated substrate is shown. **B**. TfHex active site with sulfated substrate **6** in the active site. Sodium ion penetrated in the active site from water solution is shown by yellow ball. Negatively charged amino acids close to the sulfo-group are shown and labeled. Distance from Cδ atom of Glu 332 and Glu 546 to sulfur atom of substrate is 0.537-0.602 and 0.465-0.609 nm, respectively, from Cε atom of Asp 472 to sulfur atom of substrate it is 0.619-0.819 nm. **C**. Overlay of the active site of *S. plicatus* hexosaminidase with docked sulfated compound (**6**; vivid color) and *p*NP-GlcNAc (**2**; grey). **D**. Overlay of the active site of *S. plicatus* hexosaminidase with docked *p*NP-GlcNAc-uronate (**5**; vivid) and *p*NP-GlcNAc (**2**; grey).

The sulfated substrate 6 forms 5–7 hydrogen bonds with TfHex even though the interaction with Asp 472 was lost during simulations (Figure 8A). Binding of substrates with charged groups at C-6 embodied positive electrostatic energy, making more unfavorable total free energy of binding estimated by AutoDock (Table 3) and making them poor substrates. This can be explained by the presence of Glu 332, Glu 546 and Asp 472 in the vicinity of the substrate’s C-6 atom (Figure 8B). Overall, the carbohydrates with bulky substitution at C-6 position are accepted by TfHex as substrates. However, negatively charged substitutions at C-6 atom caused lower hydrolysis rates due to the less favorable binding energy and unstable interaction with the catalytic Glu residue. Additional stability of charged groups in the active site of the fungal enzyme could be maintained by small cations, such as the sodium ion, often present in the buffer (Figure 8B). On the other hand, after the consequent molecular dynamics the affinity of the bacterial hexosaminidase to charged compounds 5 and 6 is significantly lower than to product 4, corresponding well to the negligible results of the hydrolytic reactions (Tables 1 and 2).

**Table 3:** Components of binding energy*

**Compound**	**E**_**binding**_ **, kcal/mol**	**E**_**van der Waals**_ **+ E**_**Hydrogen bond**_ **+ E**_**Desolvation Energy**_ **, kcal/mol**	**E**_**Electrostatic Energy**_ **, kcal/mol**	**E**_**Torsional Free**_ **, kcal/mol**
pNP-GlcNAc **2**	−9.47	−11.59	−0.30	2.40
pNP-GlcNAc-uronate **5**	−7.21	−11.55	1.79	2.55
pNP-GlcNAc-sulfate **6**	−7.08	−10.58	0.82	2.68

*Components of binding energies calculated for a representative snapshot from molecular dynamics run after 10 ns of simulation.

**Figure 9:**
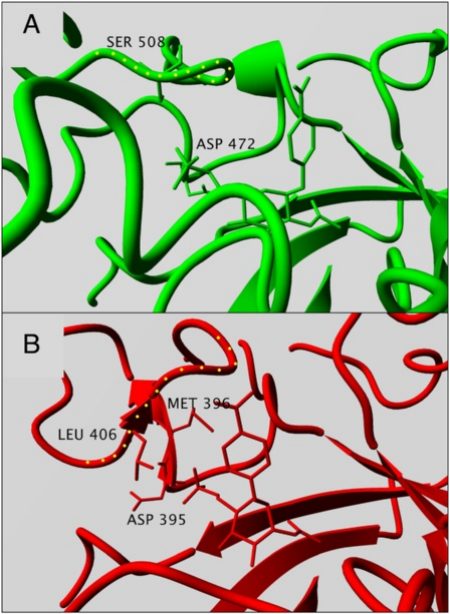
**Active site of hexosaminidases with docked** ***p*** **NP-GlcNAc-sulfate 6.** Active site of hexosaminidases with docked *p*NP-GlcNAc-sulfate **6** after molecular dynamics simulation with shown residues at the distance less than 0.3 nm from the sulfate group of the substrate. Loop 2 in the vicinity of the sulfate is marked by yellow dots: **A**. *S. plicatus* hexosaminidase **B**. TfHex.

In the beginning of the simulation of hexosaminidase from *S. plicatus* the binding energy of uronate 5 was favorable, however, during the simulation the interaction with catalytic Asp 313 and Glu 314 residues was lost. In case of the sulfated substrate 6 docking into the active site of hexosaminidase from *S. plicatus* was successful only when applying flexibility to the amino acid residues. The induced fit shifted the positions of side chains of Arg 162, Asp 395, Glu 444 and catalytic Asp 313 and Glu 314 to accommodate the sulfo-group (Figure 8). Comparison of the conformation of the bacterial and fungal hexosaminidases in proximity of the substrate C-6 atoms revealed the larger size of the fungal binding pocket as a consequence of longer loop 2 (Figure 9).

Altogether the results of molecular dynamics simulations clearly explain why the C-6 modified glycosides 5 and 6 could not be effectively hydrolyzed by the bacterial hexosaminidase. In conclusion, the difference in the affinities of the fungal and bacterial β-*N*-acetylhexosaminidases to C-6 modified substrates is determined by the divergence in the spatial orientation of loop 2, as the larger binding pocket formed by loop 2 in TfHex allows the accommodation of the bulky substituents at C-6 of the substrate (Figure 9), while the docking of such substrates in the bacterial hexosaminidase caused the distortions of active site amino acids, particularly catalytic, and hence blocked the reaction at all.

## Conclusions

In this work, the biotechnologically interesting β-*N*-acetylhexosaminidase from *Talaromyces flavus* was studied using the methods of homology modeling, molecular dynamics simulation and docking. Known structures of hexosaminidases from *Streptomyces plicatus*, *Ostrinia furnacalis* and human were used as templates for homology modeling of the catalytic subunit of our fungal enzyme with extremely broad substrate specificity. As simple homology modeling based on these known specific structures might be misleading, molecular dynamics needed to be included to account for the proper sampling of the conformational space of protein residues, especially in the binding pocket. The older models of fungal β-*N*-acetylhexosaminidases (*A. oryzae*, *P. oxalicum*) were based on the human and bacterial structures, which have lower sequence identity to fungal hexosaminidases. The recently published structure of insect hexosaminidase is the only one that shows a sequence identity higher than the generally accepted threshold for homology modeling of 30%, moreover, the orientation of the loops above the active site of the insect enzyme helped us determine the position of the edges of the loops of our studied enzyme more precisely.

Main attention in the article was focused on the structural features of β-*N*-acetylhexosaminidase from *Talaromyces flavus* leading to its unique substrate flexibility, which was demonstrated on the docking of the C-6 modified substrates *p*NP-GlcNAc-uronate and *p*NP-GlcNAc-sulfate. These glycosides were not accepted as substrates by the bacterial and human hexosaminidases, while fungal enzyme from *Talaromyces flavus* was able to catalyze both hydrolytic and transglycosylation reactions with these substrates. Surprisingly, while the specific contacts with the substrates in the binding sites are nearly identical among the hexosaminidases studied, the main difference was found in the spatial orientation of loops 1 and 2. Differences in the primary stricture of loop 2 (particularly substitution of Glu 332 by a nonpolar residue) determine better stabilization of the C-4 modified (*galacto*-) substrate 3 in the active site of fungal hexosaminidases than in bacterial hexosaminidases.

Conformation of loop 2 close to the C-6 atom of the substrates allows to accommodate larger substitution at C-6 position in hexosaminidase from *Talaromyces flavus*, but not in the other enzymes. Even though the negative charge of the substrate at this position decreases electrostatic interaction energy with the enzyme, the charge can be compensated by small positive ions from the buffer. Finally, our molecular dynamics simulations also revealed the changes in the active site after the hydrolysis of GlcNAc-glycosides leading to the easier release of the product (GlcNAc), such as rotation of the side chain of catalytic glutamate and shift of Cα-atoms of the catalytic residues.

## Methods

### Phylogenetic analysis

BLAST [30] was used for screening the non-redundant NCBI database for sequence homologs to include in the phylogenetic analysis. As a threshold for sequence acceptance a sequence coverage of greater than 50% and at least 30% sequence identity was applied. The identity threshold was decreased to 28% for bacteria to obtain a relevant number of sequences for comparison. Paralogues, hypothetical and unclassified proteins were deleted from the initial dataset. Multiple sequence alignment was performed with T-Coffee [38] and treated with JalView [39]. N- and C-terminal parts of sequences in the multiple sequence alignment were ignored for data homogeneity. For the phylogenetic analysis itself the Neighbor Joining method with Jones-Taylor-Thornton substitution matrix was used. All calculations were run in the BioNJ program [40]. To test the robustness of the tree, bootstrapping was applied with 1000 datasets to improve sampling. The consensus phylogram was generated with the TreeDyn tool [41].

### Template selection

The sequence of β-*N*-acetylhexosaminidase from *Talaromyces flavus* (TfHex) truncated at its N-terminus (without first 125 amino acid residues [GenBank:AEQ33603]) was used for BLAST search [30] in the Protein DataBank [42]. Templates applicable for homology modeling were identified: insect hexosaminidase (sequence identity 34% over 98% of sequence) [24] and human β-chain (30% identity with 99% sequence coverage) [20]. Despite its lower identity with TfHex (29%), the hexosaminidase from Streptomyces plicatus [14] was selected as the third template for homology modeling. The identities of other bacterial hexosaminidases with TfHex were less than 25%, however low identity and lack of additional information for modeling leads to rejection of them. Sequences and structures of the best fitting templates were downloaded from the Protein DataBank [42]. Secondary structure prediction for alignment guidance was done by consensus secondary structure prediction [43]. Multiple sequence structure based alignment, constructed with T-coffee [38], was manually corrected in JalView [39] based on the templates alignment done with MUSTANG algorithm implemented in YASARA [44].

### Homology modeling and refinement

A set of homology models of TfHex was prepared with Modeller [31] and the most accurate one of them was selected based on quality parameters. Quality of initial and refined models was estimated with ProSA [45], Molprobity [32] and Vadar [33]. The dimeric structure of insect hexosaminidase, in which the active site bears the inhibitor TMG-chitotriomycin and thus restricts the conformational space for loops close to the dimerization surface or the inhibitor, was used as a template for modeling the correct accommodation of the long loops in the fungal enzyme. The best obtained models of TfHex were further refined by molecular dynamics simulation with YASARA (TIP3 water model, periodic boundary condition, PME used for long-range interaction [46], YASARA 2 force field, NPT ensemble) [47]. The refinement protocol was the following: a short relaxation of water, in which the protein was constrained, was followed by molecular dynamics simulation of the system, in which all of the protein except the long loops were kept fixed for 20 ns. In the next step the simulation was continued keeping position restrains only on active site amino acid residues for additional 30 ns. The minimum energy structure from the last 5 ns of unrestrained simulation was selected to generate the final equilibrated model. The best refined model of TfHex, based on the quality assessment mentioned above, was selected and used for further molecular dynamics. The average distance between the centers of mass of the two monomers within the dimer was 3.787 nm. The buried surface area of one monomer was 21.40475 nm^2^. The number of hydrogen bonds formed between the monomers in the dimer during the period of molecular dynamics, in which the root mean square deviation reached a stable plateau was 13 to 21.

### Glycosylation and ligand docking

The refined model of TfHex was used for glycosylation by GlyProt [34]. Glycosylated and deglycosylated models with the substrate chitobiose docked in the active site were used for further molecular dynamics (water TIP3, periodic boundary condition, YASARA 2 force field, NPT ensemble) to study the effect of glycosylation of the enzyme.

Structures of carbohydrate ligands were extracted from Protein DataBank [42]: GlcNAc from structure of hexosaminidase from *S. plicatus* 1m01 [48], and chitobiose from the structure of chitobiase from *Serratia marcescens* 1qbb [19]. Other compounds were prepared and optimized in YASARA by modification of chitobiose from 1qbb [18]. YASARA 2 force-field parameters for all used compounds were assigned using the fully automatic approach provided by AutoSMILES in YASARA [49].

Local docking of the studied compounds into the active site of insect (*O. furnacalis*), bacterial (*S. plicatus*) and fungal (*T. flavus*) hexosaminidases and also binding energy calculation was done by AutoDock 4.2.3 (grid space 0.275 Å, Lamarckian genetic algorithm, number of runs 100) [50,51]. All reported results are averages from three independent molecular dynamics runs of the respective substrate-enzyme complexes and standard deviations are reported in Table 2. Each molecular dynamics simulation of enzyme-substrate complexes was run for 10 ns, including a relaxation period of 2 ns. Complexes equilibrated already after 7 ns when the root mean square deviation of C-alpha atoms reached a plateau. Information of possible rotameric conformations in the equilibrated complex was included by calculating the free energy of binding estimate as an averaged value for a number of representative structures extracted from the stable period of the respective molecular dynamics run. The free energy of binding estimated by AutoDock is calculated according to the following equations (1) and (2), as defined in AutoDock documentation:

1 $$ {\mathrm{E}}_{\mathrm{Free}\ \mathrm{Energy}\ \mathrm{of}\ \mathrm{Binding}}={\mathrm{E}}_{\mathrm{Final}\ \mathrm{Intermolecular}}+{\mathrm{E}}_{\mathrm{Final}\ \mathrm{Total}\ \mathrm{Internal}}+{\mathrm{E}}_{\mathrm{Torsional}\ \mathrm{Free}}-{\mathrm{E}}_{\mathrm{Unbound}\ \mathrm{System}\hbox{'}\mathrm{s}\ \mathrm{Energy}} $$

2 $$ {\mathrm{E}}_{\mathrm{Final}\ \mathrm{Intermolecular}}={\mathrm{E}}_{\mathrm{Van}\ \mathrm{d}\mathrm{e}\mathrm{r}\ \mathrm{Waals}}+{\mathrm{E}}_{\mathrm{Hydrogen}\ \mathrm{bond}}+{\mathrm{E}}_{\mathrm{Desolvation}\ \mathrm{Energy}}+{\mathrm{E}}_{\mathrm{E}\mathrm{lectrostatic}\ \mathrm{Energy}} $$

Enzyme-substrate simulations were performed and analyzed with YASARA. Interactions in the active site were shown with LigPlot + [52].

## Additional file

Additional file 1: **Supplementary materials.** More details on model refinement, sequence alignment and substrate-enzyme interaction.

## Abbreviations

*p*NP-GlcNAc: 4-nitrophenyl 2-acetamido-2-deoxy-β-D-glucopyranoside
*p*NP-GalNAc: 4-nitrophenyl 2-acetamido-2-deoxy-β-D-galactopyranoside
*p*NP-GlcNAc-uronate: 4-nitrophenyl 2-acetamido-2-deoxy-β-D-glucopyranosiduronic acid
*p*NP-GlcNAc-sulfate: 4-nitrophenyl 2-acetamido-2-deoxy-6-*O*-sulfo-β-D-glucopyranoside
GlcNAc: 2-acetamido-2-deoxy-D-glucopyranose
NAG-thiazoline: 2′-methyl-2-acetamido-2-deoxy-α-D-glucopyranosyl-[2,1-*d*]-∆2′-thiazoline
TfHex: β-*N*-acetylhexosaminidase from *Talaromyces flavus*

## References

[1] Slámová K, Bojarová P, Petrásková L, Křen V (2010). β-*N*-Acetylhexosaminidase: What’s in a name…?. Biotechnol Adv.

[2] Crout DHG, Singh S, Swoboda BEP, Critchley P, Gibson WT (1992). Biotransformation in carbohydrate synthesis. *N*-Acetylgalactosaminyl transfer on to methyl *N*-acetyl-β-D-glucosaminide (methyl 2-acetamido-2-deoxy-α-D-glucopyranoside) catalysed by a β-N-acetylgalactosaminidase from *Aspergillus oryzae*. J Chem Soc, Chem Commun.

[3] Carmona T, Fialová P, Křen V, Ettrich R, Martínková L, Moreno-Vargas AJ (2006). Cyanodeoxy-glycosyl derivatives as substrates for enzymatic reactions. Eur J Org Chem..

[4] Fialová P, Weignerová L, Rauvolfová J, Přikrylová V, Pišvejcová A, Ettrich R (2004). Hydrolytic and transglycosylation reactions of *N*-acyl modified substrates catalysed by β-*N*-acetylhexosaminidases. Tetrahedron..

[5] Ogata M, Zeng X, Usui T, Uzawa H (2007). Substrate specificity of *N*-acetylhexosaminidase from *Aspergillus oryzae* to artificial glycosyl acceptors having various substituents at the reducing ends. Carbohydr Res..

[6] Uzawa H, Zeng X, Minoura N (2003). Synthesis of 6′-sulfodisaccharides by β-*N*-acetylhexosaminidase-catalyzed transglycosylation. Chem Commun..

[7] Bojarová P, Slámová K, Křenek K, Gažák R, Kulik N, Ettrich R (2011). Charged hexosaminides as new substrates for β-*N*-acetylhexosaminidase-catalyzed synthesis of immunomodulatory disaccharides. Adv Synth Catal.

[8] Slámová K, Gažák R, Bojarová P, Kulik N, Ettrich R, Pelantová H (2010). 4-Deoxy-substrates for β-*N*-acetylhexosaminidases: How to make use of their loose specificity. Glycobiology..

[9] Plíhal O, Sklenář J, Hofbauerová K, Novák P, Man P, Pompach P (2007). Large propeptides of fungal β-*N*-acetylhexosaminidases are novel enzyme regulators that must be intracellularly processed to control activity, dimerization, and secretion into the extracellular environment. Biochemistry..

[10] Plíhal O, Sklenář J, Kmoníčková J, Man P, Pompach P, Havlíček V (2004). *N*-Glycosylated catalytic unit meets *O*-glycosylated propeptide: complex protein architecture in a fungal hexosaminidase. Biochem Soc Trans..

[11] Vaněk O, Brynda J, Hofbauerová K, Kukačka Z, Pachl P, Bezouška K (2011). Crystallization and diffraction analysis of β-*N*-acetylhexosaminidase from *Aspergillus oryzae*. Acta Cryst..

[12] Ettrich R, Kopecký V, Hofbauerová K, Baumruk V, Novák P, Pompach P (2007). Structure of the dimeric N-glycosylated form of fungal β-N-acetylhexosaminidase revealed by computer modeling, vibrational spectroscopy, and biochemical studies. BMC Struct Biol..

[13] Ryšlavá H, Kalendová A, Doubnerová V, Skočdopol P, Kumar V, Kukačka Z (2011). Enzymatic characterization and molecular modeling of an evolutionary interesting fungal β-*N*-acetylhexosaminidase. FEBS J..

[14] Mark BL, Vocadlo DJ, Zhao D, Knapp S, Withers SG, James MN (2001). Biochemical and structural assessment of the 1-*N*-azasugar GalNAc-isofagomine as a potent family 20 β-*N*-acetylhexosaminidase inhibitor. J Biol Chem..

[15] Sumida T, Ishii R, Yanagisawa T, Yokoyama S, Ito M (2009). Molecular cloning and crystal structural analysis of a novel β-*N*-acetylhexosaminidase from *Paenibacillus sp.* TS12 capable of degrading glycosphingolipids. J Mol Biol.

[16] Jiang YL, Yu WL, Zhang JW, Frolet C, Di Guilmi AM, Zhou CZ (2011). Structural basis for the substrate specificity of a novel β-*N*-acetylhexosaminidase StrH protein from *Streptococcus pneumoniae* R6. J Biol Chem..

[17] Langley DB, Harty DW, Jacques NA, Hunter N, Guss JM, Collyer CA (2008). Structure of *N*-acetyl-β-D-glucosaminidase (GcnA) from the endocarditis pathogen *Streptococcus gordonii* and its complex with the mechanism-based inhibitor NAG-thiazoline. J Mol Biol..

[18] Tews I, Perrakis A, Oppenheim A, Dauter Z, Wilson KS, Vorgias CE (1996). Bacterial chitobiase structure provides insight into catalytic mechanism and the basis of Tay-Sachs disease. Nature Struct Biol..

[19] Ramasubbu N, Thomas LM, Ragunath C, Kaplan JB (2005). Structural analysis of dispersin B, a biofilm-releasing glycoside hydrolase from the periodontopathogen *Actinobacillus actinomycetemcomitans*. J Mol Biol..

[20] Mark BL, Mahuran DJ, Cherney MM, Zhao D, Knapp S, James MN (2003). Crystal structure of human β-hexosaminidase B: understanding the molecular basis of Sandhoff and Tay-Sachs disease. J Mol Biol..

[21] Lemieux MJ, Mark BL, Cherney MM, Withers SG, Mahuran DJ, James MN (2006). Crystallographic structure of human β-hexosaminidase A: interpretation of Tay-Sachs mutations and loss of GM2 ganglioside hydrolysis. J Mol Biol..

[22] Maier T, Strater N, Schuette CG, Klingenstein R, Sandhoff K, Saenger W (2003). The X-ray crystal structure of human β-hexosaminidase B provides new insights into Sandhoff disease. J Mol Biol..

[23] Bateman KS, Cherney MM, Mahuran DJ, Tropak M, James MN (2011). Crystal structure of β-hexosaminidase B in complex with pyrimethamine, a potential pharmacological chaperone. J Med Chem.

[24] Liu T, Zhang H, Liu F, Wu Q, Shen X, Yang Q (2011). Structural determinants of an insect β-*N*-acetyl-D-hexosaminidase specialized as a chitinolytic enzyme. J Biol Chem..

[25] Liu T, Zhang H, Liu F, Chen L, Shen X, Yang Q (2011). Active-pocket size differentiating insectile from bacterial chitinolytic β-*N*-acetyl-D-hexosaminidases. Biochem J..

[26] Tews I, Terwisscha van Scheltinga AC, Perrakis A, Wilson KS, Dijkstra BW (1997). Substrate-assisted catalysis unifies two families of chitinolytic enzymes. J Am Chem Soc.

[27] Mark BL, Vocadlo DJ, Knapp S, Triggs-Raine BL, Withers SG, James MN (2001). Crystallographic evidence for substrate-assisted catalysis in a bacterial β-hexosaminidase. J Biol Chem..

[28] Slámová K, Bojarová P, Gerstorferová D, Fliedrová B, Hofmeisterová J, Fiala M (2012). Sequencing, cloning and high-yield expression of a fungal β-*N*-acetylhexosaminidase in *Pichia pastoris*. Protein Expr Purif..

[29] Kulik N, Slámová K (2011). Computational modelling of catalytic properties and modified substrates of fungal β-*N*-acetylhexosaminidases. Mini-Rev Org Chem..

[30] Altschul SF, Gish W, Miller W, Myers EW, Lipman DJ (1990). Basic local alignment search tool. J Mol Biol..

[31] Sali A, Blundell TL (1993). Comparative protein modelling by satisfaction of spatial restraints. J Mol Biol..

[32] Davis IW, Leaver-Fay A, Chen VB, Block JN, Kapral GJ, Wang X (2007). MolProbity: all-atom contacts and structure validation for proteins and nucleic acids. Nucleic Acids Res..

[33] Willard L, Ranjan A, Zhang H, Monzavi H, Boyko RF, Sykes BD (2003). VADAR: a web server for quantitative evaluation of protein structure quality. Nucleic Acids Res..

[34] Bohne-Lang A, von der Lieth CW (2005). GlyProt: *in silico* glycosylation of proteins. Nucleic Acids Res..

[35] Pace CN, Shirley BA, McNutt M, Gajiwala K (1996). Force contribution to the conformational stability of protein. FASEB J..

[36] Weignerová L, Vavrušková P, Pišvejcová A, Thiem J, Křen V (2003). Fungal β-N-acetylhexosaminidases with high β-N-acetylgalactosaminidase activity and their use for syntheis of β-GalNAc-containing oligosaccharides. Carbohydr Res.

[37] Liu T, Zhou Y, Chen L, Chen W, Liu L, Shen X (2012). Structural insights into cellulolytic and chitinolytic enzymes revealing crucial residues of insect β-*N*-acetyl-D-hexosaminidase. PLoS One.

[38] Notredame C, Higgins DG, Heringa J (2000). T-coffee: a novel method for fast and accurate multiple sequence alignment. J Mol Biol..

[39] Waterhouse AM, Procter JB, Martin DMA, Clamp M, Barton GJ (2009). Jalview Version 2 - a multiple sequence alignment editor and analysis workbench. Bioinformatics..

[40] Gascuel O (1997). BIONJ: an improved version of the NJ algorithm based on a simple model of sequence data. Mol Biol Evol..

[41] Chevenet F, Brun C, Banuls AL, Jacq B, Chisten R (2006). TreeDyn: towards dynamic graphics and annotations for analyses of trees. BMC Bioinformatics..

[42] Bernstein FC, Koetzle TF, Williams GJB, Meyer EF, Brice MD, Rodgers JR (1977). The protein data bank: a computer-based archival file for macromolecular structures. J Mol Biol..

[43] Combet C, Blanchet C, Geourjon C, Deléage G (2000). NPS@: network protein sequence analysis. Trends Biochem Sci..

[44] Konagurthu AS, Whisstock JC, Stuckey PJ, Lesk AM (2006). MUSTANG: a multiple structural alignment algorithm. Proteins..

[45] Wiederstein M, Sippl MJ (2007). ProSA-web: interactive web service for the recognition of errors in three-dimensional structures of proteins. Nucleic Acids Res..

[46] Essman U, Perera L, Berkowitz ML, Darden T, Lee H, Pedersen LG (1995). A smooth particle mesh Ewald method. J Chem Phys..

[47] Krieger E, Koraimann G, Vriend G (2002). Increasing the precision of comparative models with YASARA NOVA - a self-parameterizing force field. Proteins..

[48] Williams SJ, Mark BL, Vocadlo DJ, James MN, Withers SG (2002). Aspartate 313 in the *Streptomyces plicatus* hexosaminidase plays a critical role in substrate-assisted catalysis by orienting the 2-acetamido group and stabilizing the transition state. J Biol Chem..

[49] Jakalian A, Jack DB, Bayly CI (2002). Fast, efficient generation of high-quality atomic charges. AM1-BCC model: II. Parameterization and validation. J Comput Chem.

[50] Morris GM, Goodsell DS, Halliday RS, Huey R, Hart WE, Belew RK (1998). Automated docking using a Lamarckian genetic algorithm and empirical binding free energy function. J Comput Chem..

[51] Morris GM, Huey R, Lindstrom W, Sanner MF, Belew RK, Goodsell DS (2009). Autodock4 and AutoDockTools4: automated docking with selective receptor flexibility. J Comput Chem..

[52] Laskowski RA, Swindells MB (2011). LigPlot+: multiple ligand-protein interaction diagrams for drug discovery. J Chem Inf Model..

