# Supplementary material for: Computational study of β-N-acetylhexosaminidase from Talaromyces flavus, a glycosidase with high substrate flexibility
Source: BMC Bioinformatics. 2015 Jan 28;16:28. doi: 10.1186/s12859-015-0465-8 (PMC4384365; doi:10.1186/s12859-015-0465-8)
Supplement: Supplementary file 1 — Supplementary materials. More details on model refinement, sequence alignment and substrate-enzyme interaction. [file 12859_2015_465_MOESM1_ESM.pdf]

Supplementary materials

Computational study of β-*N*-acetylhexosaminidase from *Talaromyces flavus*, a glycosidase with high substrate flexibility

Natallia Kulik [1,], Kristýna Slámová [2], Rüdiger Ettrich [1,3], Vladimír Křen [2]*

[1]Department of Structure and Function of Proteins, Institute of Nanobiology and Structural Biology of GCRC, Academy of Sciences of the Czech Republic, Zamek 136, 37333 Nove Hrady, Czech Republic

[2]Laboratory of Biotransformation, Institute of Microbiology, Academy of Sciences of the Czech Republic, Videnska 1083, 14220 Praha 4, Czech Republic

[3]Faculty of Sciences, University of South Bohemia in Ceske Budejovice, Zamek 136, 37333 Nove Hrady, Czech Republic

*Corresponding author: Kulik Natallia, Institute of Nanobiology and Structural Biology of GCRC, Academy of Sciences of the Czech Republic, Zamek 136, 373 33 Nove Hrady, Czech Republic, phone: +420 775 133 670, e-mail: kulik@nh.cas.cz.

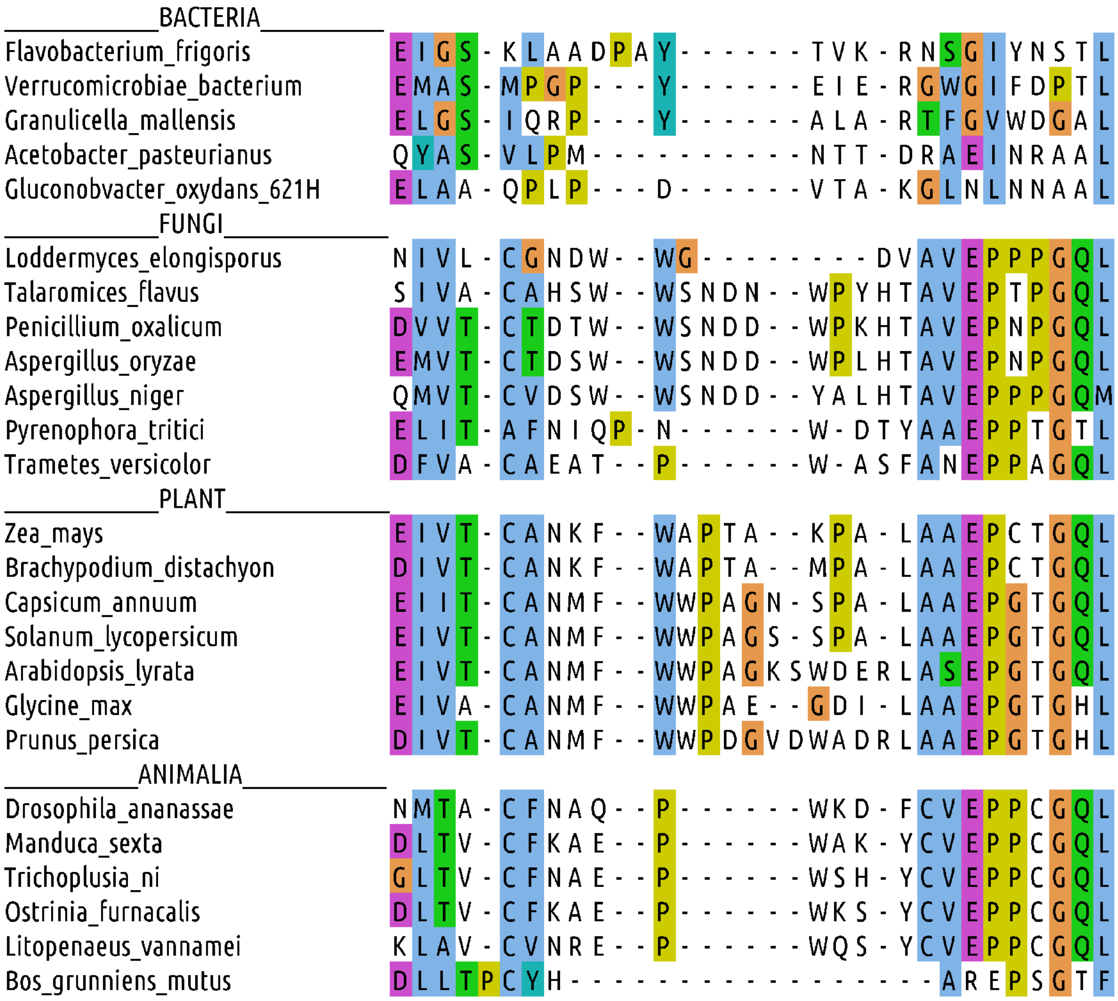

Figure S1. Part of multiple sequence alignment containing loop 1 used for phylogenetic analysis. Amino acid composition of loop 1 in plants and fungi is similar and rich in aromatic residues and proline, so we could expect that they will have also similar conformation. In the hexosaminidases from *Aspergillus oryzae* and *Penicillium oxalicum* this loop has the same size and similar amino acid composition as the hexosaminidase from *Talaromyces flavus* (TfHex).

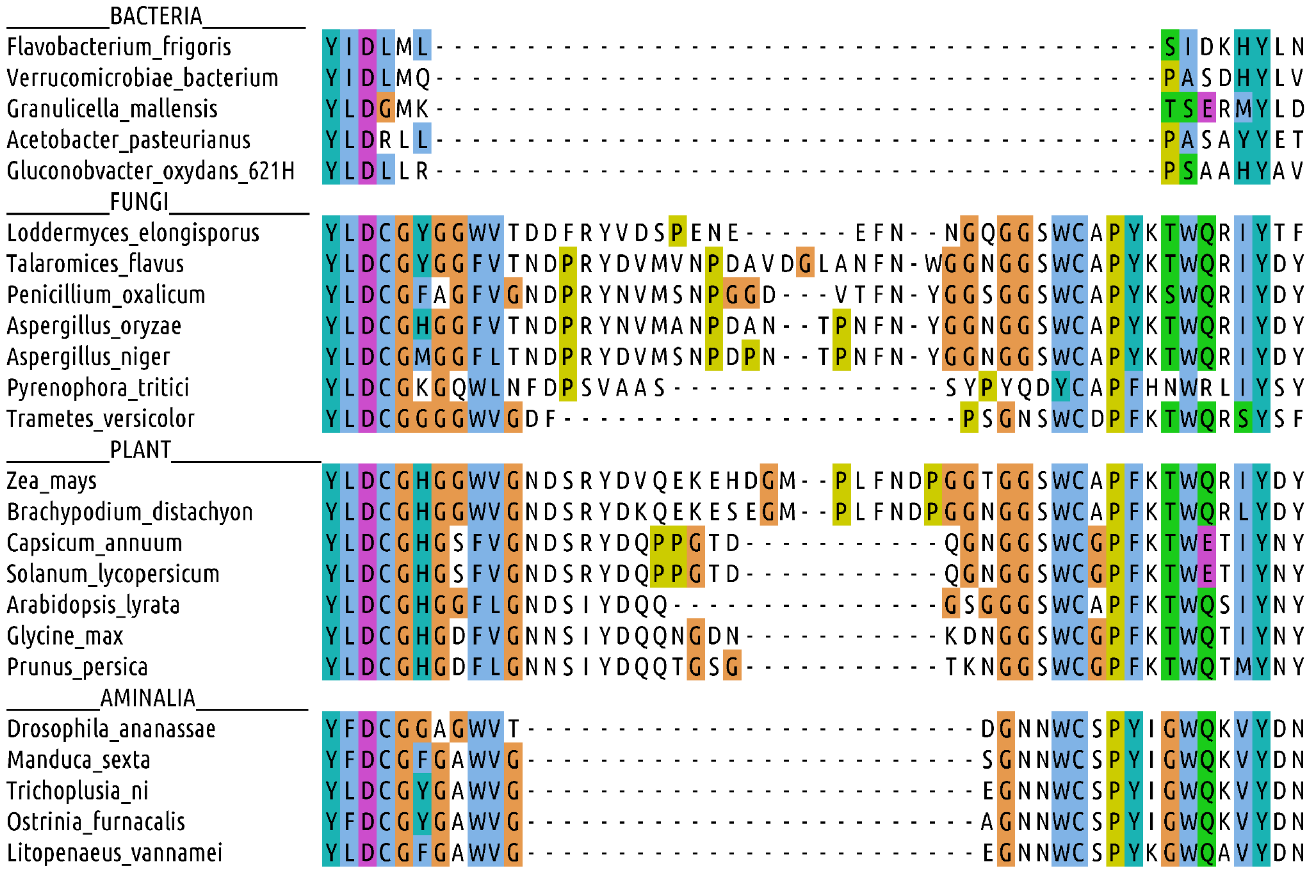

Figure S2. **Part of multiple sequence alignment containing loop 2 used for phylogenetic analysis.** Loops 1 and 2 are longest in plant (especially in *Arabidopsis lyrata* and *Prunus persica*) and in fungal sequences. N-terminal and C-terminal ends of loop 1 and loop 2 have similar amino acid composition in animals, fungi and plants.

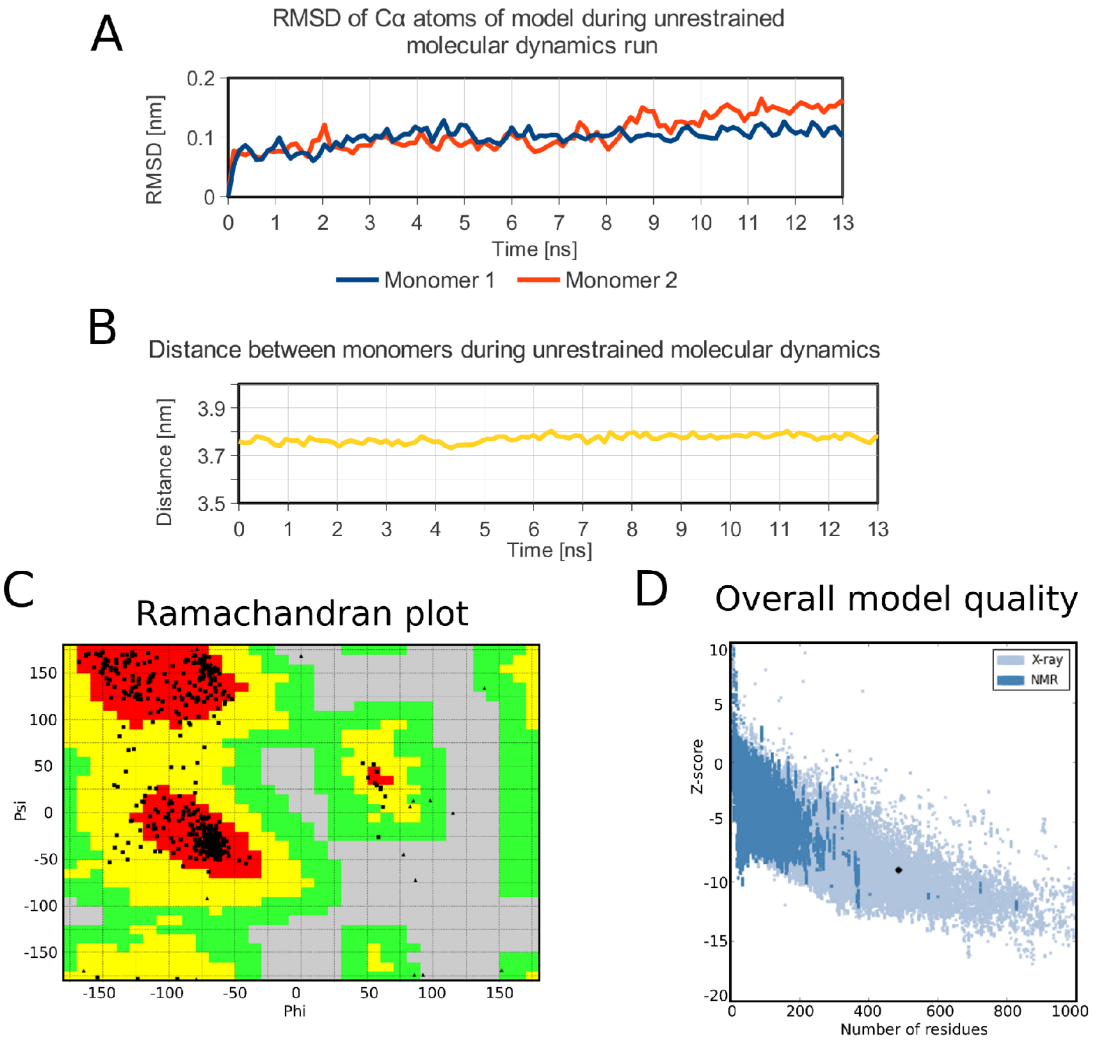

Figure S3. Statistics of the refinement of TfHex model.

A. Root means square deviation (RMSD) of Cα atoms of monomers. **B.** Distance between monomers in dimer. **C.** Ramachandran plot. The red, yellow and green regions correspond to the favored, allowed, and "generously allowed" regions, grey color fields are assumed as disallowed for phi/psy backbone angles. **D.** Protein quality analyzed by ProSA. ProSA z-score is -9.06, that is fitted in region of values found for structures solved by X-ray crystallography. Knowledge-based potential of mean force averaged over 40 residues is negative for all residues, except for the region of loop 2, which could be connected to its high flexibility and also absence of a suitable template.

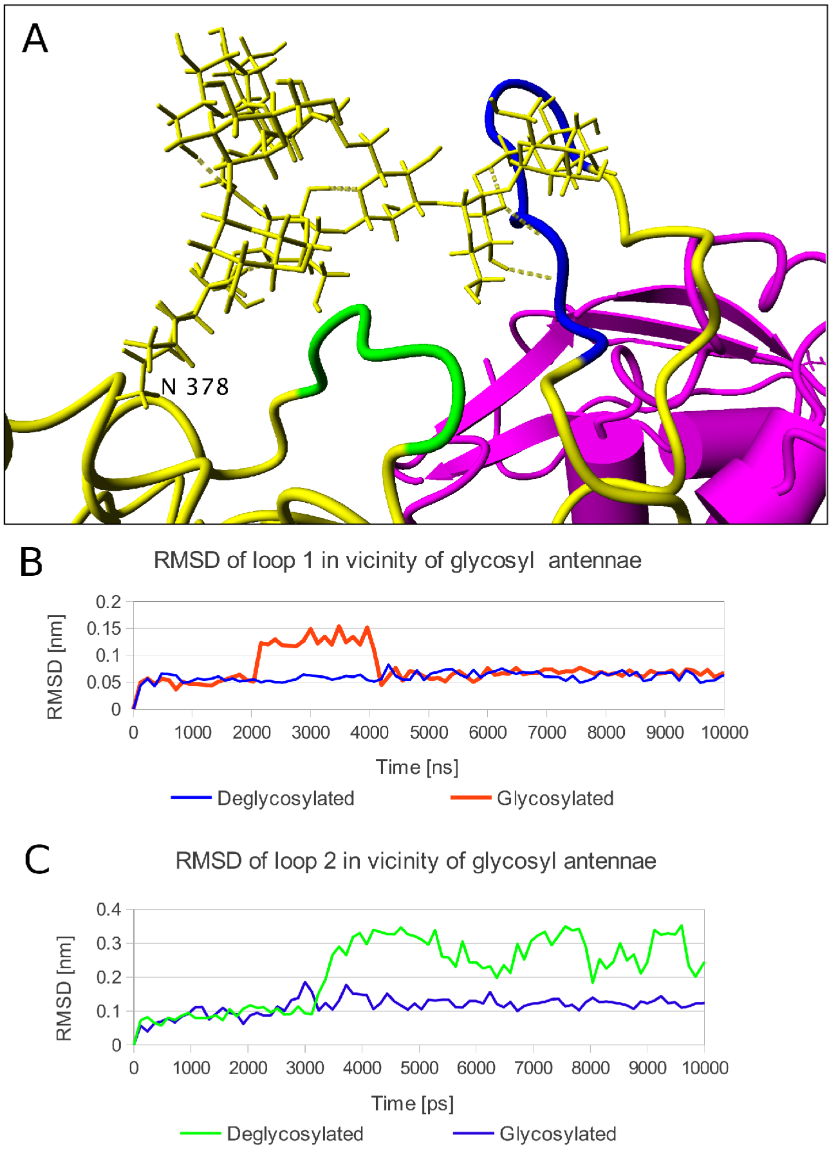

Figure S4. Glycosylated TfHex.

A. Part of model of hexosaminidase from *T. flavus* with shown glycosyl antennae at Asn 378, partly covering loop 1 (green) and interacting with loop 2 (blue). One monomer is shown by yellow color, another is magenta, glycan chain is shown in stick representation, hydrogen bonds are marked by yellow dotted lines. **B.** and **C**. Root means square deviation of residues of loop 1 (**B**) and loop 2 (**C**) in the vicinity of the glycan at Asn 378 (threshold was 0.3 nm for distance from carbohydrates).

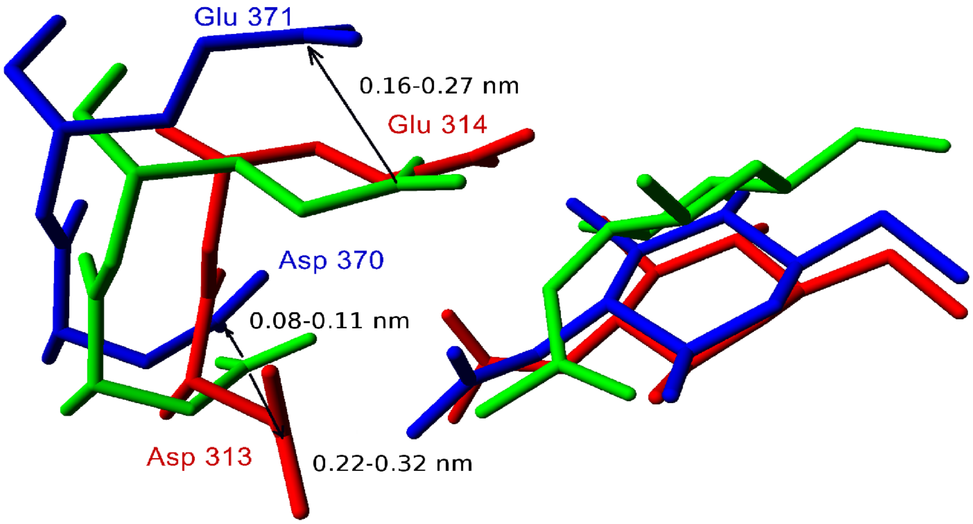

Figure S5. Overlay of hexosaminidases with docked product GlcNAc.

Position of product (2-acetamido-2-deoxy-D-glucopyranose) in the active sites of hexosaminidase from *T. flavus* before molecular dynamics simulation (green) and after (red) and hexosaminidase from *S. plicatus* after molecular dynamics (blue) is shown extracted from representative molecular dynamics run. Initial position of catalytic residues in *S. plicatus* hexosaminidase is similar to *T. flavus* enzyme and hence not shown. Change in the position of the corresponding atoms of catalytic residues is highlighted by arrows with distances in nanometers, calculated from independent molecular dynamis run for a stable period.

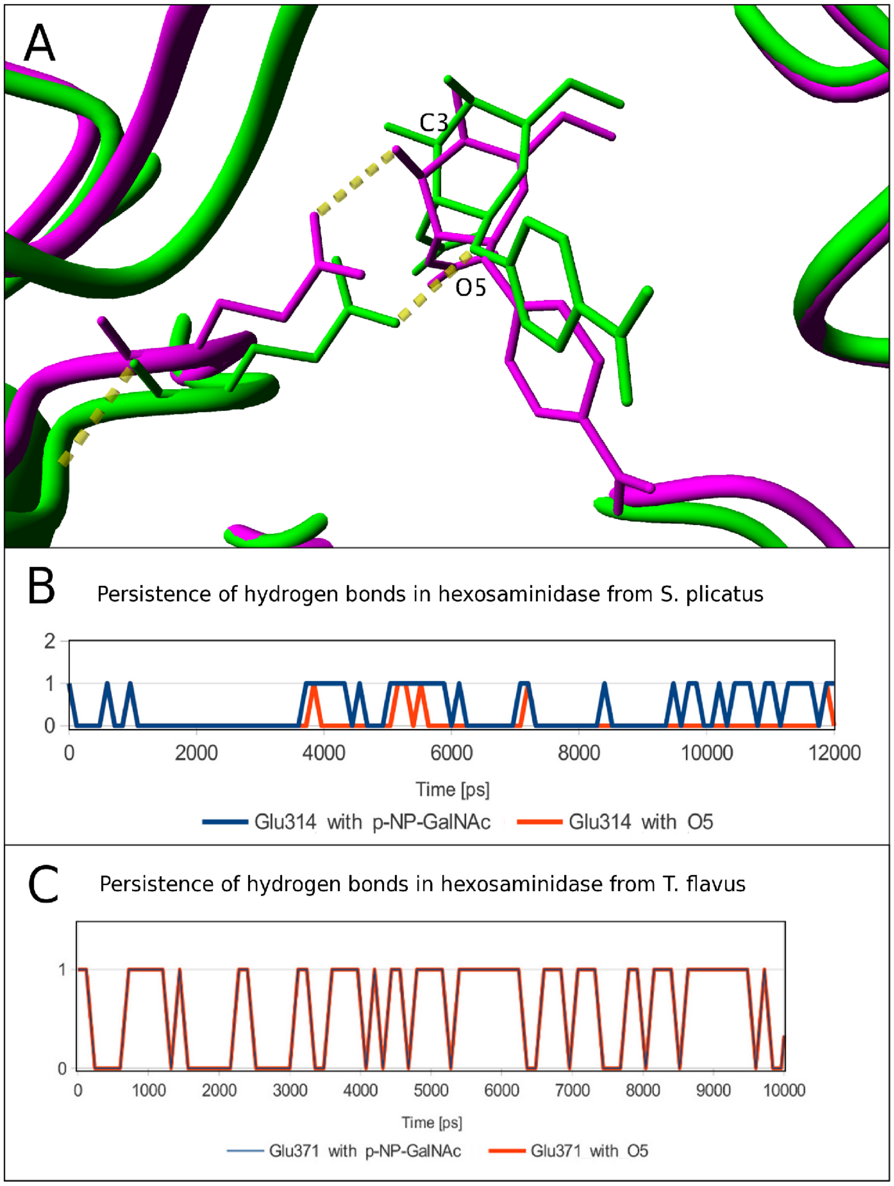

Figure S6. Change in the hydrogen bonding interaction of catalytic glutamic residue.

A. Complex of *p*NP-GlcNAc with *S. plicatus* hexosaminidase in the beginning of simulation is green, snapshot of this complex after 10 ns molecular dynamics is magenta. Loops close to the active site are shown like tubes, Glu 314 and 4-nitrophenyl 2-acetamido-2-deoxy-β-D-glucopyranoside as sticks, the rest of the protein and hydrogen atoms are hidden. **B.** and **C.** Formation of hydrogen bonds during molecular dynamics: “0” means absence of interaction, “1” means presence of hydrogen bond.
